# 

**Published:** 1898-05

**Authors:** 


### THE
### AMERICAN JOURNAL
OF
### DENTAL SCIENCE,

EDITED BY

F. J. S. GORGAS, M. D., D. D. S.

RICHARD GRADY, M. D., D. D. S.

VOLUME XXXII—THIRD SERIES.

LIBRARY
SURGEON GENERAL'S OFFICE
FEB. 11.-1901
168865.

BALTIMORE:
SNOWDEN & COWMAN MFG. CO.

LONDON:
TRUBNER & CO., PATERNOSTER ROW.

1899.

### Index to Vol. XXXII---Third Series.

A Clinic on a Fusible Alloy.................................... 12
Amalgam.................................................. 31
A Mysterious Stain........................................ 45
A New Function of the Antrum............................ 93
A Discussion Following the Reading of a Paper upon "Syphilitic Affections of the Mouth"........................ 63
Adapting Artificial Dentures............................. 109
Advertising................................................ 120
A Student's Damage Suit.................................. 143
Adaptation and Retention of Dentures.................... 163
Alcohol as a Disinfectant................................. 190
A Dentist.................................................. 192
A Few Dental Points....................................... 269
Amalgam Stain............................................. 288
An Important Dento-Legal Function....................... 372
A Dictionary of Dental Science........................... 375
A Good Cement............................................. 381
A Cheap Method of Crowning.............................. 382
A New Army Hospilal...................................... 429
A Few Observations....................................... 456
A New Counter-Die........................................ 474
An Exact Method for Measuring Rubber................... 477
A Dentist for the Army.................................... 478
Artifieial Dentures in their Relation to the Speaking or the Singing Voice........................................ 491
Anesthesia by Rapid Breathing............................ 498
An Easy Method of Refining Gold......................... 503
A New Dentinal Obtundent................................ 510
Alloy as a Filling Material............................... 518
A New Way of Preserving Meat............................ 523
Annealing Gold............................................ 528
A Last Word............................................... 529
A Tube-Dowel Crown....................................... 574
Bites and Some Methods of Taking Them................... 214
Bleeding Gums............................................. 426
Baltimore College of Dental Surgery Commencement....... 571
Caries vs. Erosion........................................ 42
Caoutchouc and Gutta-Percha Cements..................... 88
Condensation of Gold Fillings............................ 103
Care and Treatment of Children's Teeth................... 107
Cohesive and Non-Cohesive Gold.......................... 141
Clinical Report on Method of Operation.................. 195

Cardinal Points in the Successful Insertion of Pemanent Fillings........ 241
Cement........ 286
Carious Dentine........ 288
Cataphoresis........ 289
Causes of Dental Caries and Prophylaxis........ 308
Carious Dentin........ 330
Counting Blood Corpuscles........ 333
Celluloid Matrices........ 335
Committees of the National Dental Association........ 376
Carious Dentin........ 384
Cataphoresis—A Warning........ 423
Contouring........ 479
Continuous-Gum Work........ 537
Dentistry in the Twenties........ 23
Dishonest Dentistry........ 40
Dr. A. J. Volck's Seventieth Birthday........ 49
Dr. Evans........ 78
Dentists' Convention........ 85
Dr. A. J. Volck's Seventieth Birthday. A Characteristic Event........ 90
Danger from Air Injection........ 96
Dental Anatomy........ 97
Dental Ethics........ 115
Death from Hodgkins' Disease........ 142
Dental Ethics........ 156
Dental Technic........ 225
Destruction of Pulp and Gold Crown by Mercuric Action Within Amalgam........ 238
Dr. Gardner Quincy Colton........ 278
Death Rate of Dentists........ 281
Disease of the Peridental Membrane........ 320
Destroying Pulps Without Arsensic........ 333
Display of Gold in Teeth........ 371
Dr. Black's Theory........ 431
Dental Fees........ 432
Degeneration and Decay of the Oral Tissues from Lack of Exercise........ 512
Dental Detection of Criminals........ 524
Dental Education of the Public........ 548
Dental Department University of Maryland Commencement. 569
Death From Cutting a Wisdom Tooth........ 573
Extraction While Swelling of Gums and Toothache........ 150
Education and Starvation........ 190
Experiments With Alloys........ 276
Exposed Pulps........ 414

Extracts from an Article upon "Some Overlooked Sources of Infection..... 425
Eucaine..... 432
Exposing Fraudulent Colleges..... 461
Education of the Public..... 546
Ethyl Bromide as an Anæsthetic..... 560
Floss Silk..... 94
Filling Materials..... 95
Foreign Doctors and Dentists in Italy..... 144
Fees, and How to Collect Them..... 254
Filling the Upper Bicuspids..... 285
For Polishing Plate Work..... 336
Frequency of Dental Caries in Children..... 364
Filling with Gold over Cement..... 378
Fitting Porcelain Inlays..... 384
Filling vs. Crown..... 526
Gingivitis and its Relation to Crown Work..... 129
Germicides and Antiseptics in Dentistry..... 175
Getting the Bite..... 240
Gold Shell Crown..... 330
Gutta-Percha as a Retainer of Crowns and Bridges..... 335
Hydronaphthol—With Suggestions for Its Use in the Treatment of the Dental Pulp, Alive or Dead..... 79
How Shall we Best Insert a Gold Filling?..... 153
Hydronaphthol in the Treatment of Dental Pulps..... 191
How to Succeed..... 279
Hardened and Washable Plaster-of-Paris..... 280
Hemorrhage After Extraction..... 376
Harder than a Diamond..... 429
Hemorrhage After Extraction..... 535
Immediate Investing Material..... 46
Ichthyol..... 331
Incipient Decay..... 411
Is Prosthetic Dentistry Advancing as it Should..... 453
Instruments to Carry Outside..... 522
In Taking..... 528
Infection of the Facial and Cervical Lymphatic Ganglia as a Result of Dental Lesions..... 557
Keep Thinking..... 467
Karolit..... 576.
Little Things in Great Things..... 33
Liquefied Air..... 39
List of Commiitee's for the Joint Meeting of the Southern Branch of the National Dental Association and the Louisiana State Dental Society..... 185
Lancing Abscess..... 476

Method of Shortening the Period of Pain During the Action of Arsenical Applications .......... 5
Meeting of American Medical Publishers' Association .......... 38
Mixiug Amalgam .......... 47
Model for Partials .......... 286
Mercury to Purify .......... 383
Matrices .......... 428
Matrices .......... 479
New Jersey State Dental Society .......... 36
Not Hart .......... 335
Natural Law and Its Penalties .......... 377
National School of Dental Technics .......... 381
New Method of Disinfection .......... 422
Nitrate of Silver .......... 458
Nature's Methods of Defense .......... 486
Opening the Bite with Cap Fillings, Preserving the Vitality of the Pulp .......... 44
On Amalgam Fillings .......... 231
One Way to Make a Gold Crown .......... 284
Oil of Cinnamon .......... 288
Operative and Prosthetic Pointers .......... 334
On Structural Changes in Human Enamel—With Special Reference to Clinical Observations on Hard and Soft Enamel .......... 337
Our Relations with our American Confreres Living and Practicing Abroad .......... 481
Pathologic Conditions of the Pharynx and Contiguous Structures During Early Childhood, Prime Factors in the Etiology of Malformed Maxillæ and Irregular Teeth, Etc. .......... 1
Pyorrhea Alveolaris .......... 42
Prosthesis .......... 72
Physiological Testing of Drugs .......... 77
Painless Dentistry .......... 114
Practical Points on Porcelain Fillings .......... 145
Prescription Writing .......... 191
Prosthetic Dentistry .......... 222
Pyorrhea .......... 287
Pyorrhea Alveolaris—With Some Notes From the Practice of the Author .......... 314
Pointing Instruments .......... 326
Professional Recruits .......... 328
Pyorrhea .......... 336
Pain and the Surgeon .......... 380
Preservation of Ether .......... 382
Plaster Impressions and Models .......... 382
Pyorrhœa Alveolaris—Personal Experience in Treatment .......... 394

Pyorrhea .......... 480
Questions Given by Maryland State Board of Dental Examiners, May. 1898 .......... 138
Remarks of Dr. E. E. Cruzen .......... 92
Reproductions of Gum Tissue .......... 110
Responsibility in Administering an Anesthetic .......... 193
Rinsing the Mouth .......... 287
Reciprocity .......... 368
Reflected Light for the Operating Chair .......... 383
Repairing Gold Fillings .......... 475
Removal of Broken Nerve B oaches and Drills From Root Canals .......... 477
Relieving Carbolic Acid Burns .......... 478
Roots and Bridges .......... 527
Report of Case of Apparent Disease of Antrum of Highmore. 542
Removal of Green Stain .......... 573
Some Failures Attending the Use of Weld's Chemico-Metallic Method of Filling Root Canals .......... 22
Saucer-Shaped Cavities .......... 44
State Dental Association .......... 92
Seventh Annual Commencement, Dental Department, University of Buffalo .......... 137
Silver Nitrate in the Treatment of Pulpless Teeth .......... 172
Shrinkage of Amalgams .......... 240
Success .......... 264
Soldering Teeth .......... 332
Soap on Dam and Disk .......... 334
Secrets and Patents Versus Professional Progress .......... 354
Sandpaper Your Joints .......... 384
Suppurative Inflammation of the Gums and Absorption of the Gums and the Alveolar Process .......... 401
Some Properties of Vulcanite Rubber .......... 419
Sun Baths .......... 431
Sterilizing Instruments .......... 431
Soldering Gold Crowns .......... 432
Setting of Crowns .......... 475
Surgical Treatmant of Abscess .......... 526
Soak it .......... 528
Seventh District Dental Society of New York .......... 568
The Drug Habit .......... 7
The National Dental Association .......... 37
The Chicago Dental Association .......... 38
To Painlessly Cure a Corn .......... 45
The Use of Non-Cohesive Gold .......... 46
Taking Difficult Impressions .......... 47
The Relative Value of Fillings .......... 75

The Care of Children's Teeth ..... 165
The Extraction of Teeth ..... 169
The Carborundum Industry ..... 188
The Mouth is the Via Natura for the Entrance of Disease ..... 198
The Preparation of the Mouth for a Denture ..... 204
The First Subscribers to the First Dental Journal ..... 232
The Patent Bill ..... 238
Tin ..... 239
The Therapeutic Treatment of Infected Tooth Root-Canals ..... 246
Tinfoil Matrices ..... 252
The Relation of Teeth to the Lips and Face ..... 258
To Duplicate Models and Impressions ..... 271
To Remove Enamel Previous to Crowning ..... 282
Tempering Instruments ..... 283
The Importance of Establishing a Technic as well as Literary Standard for College Entrance ..... 302
Taking Impressions ..... 330
The Best Styptic ..... 331
Trimming Vulcanite ..... 333
To give Fine Finish to Gold ..... 334
To Deposit Paste in Cavity ..... 336
The Dentist ..... 349
The Prolongation of Nitrous Oxid Anesthesia ..... 361
The Physical Properties of Dental Amalgams ..... 366
Treating Foul Pulp Canals ..... 383
To Solder Cap on Gold Crowns ..... 383
The Practical Value of Chemistry In Dentistry ..... 385
Treatment of the Cervical Border ..... 415
The Chicago Dental Society ..... 421
Transplanting—Resetting ..... 424
Tincture of Iodine for the Removal of Deposits ..... 426
To Fold Gold Foil Without Contact of the Fingers ..... 427
Treatment for Exposed Dentin ..... 428
The Education of the Dental Surgeon ..... 433
The Jenkins System of Porcelain Fillings ..... 437
Treatment of Cervical Borders ..... 449
To Make a Duplicate of a Denture ..... 471
Tipping Teeth ..... 476
The Medical Treatment of Toothache ..... 476
The Use of Formalin ..... 500
The Importance and Manner of Sterilization of Dental Instruments ..... 505
Thoughtless Mothers ..... 524
Treatment of Decalcified Dentine in Bottom of Cavity ..... 527
To Remove Plaster from Vulcanite Plates ..... 528
The Medicinal Prevention of Dental Decay ..... 550
Temporary Sets of Teeth ..... 575
Use of Screws ..... 16
Using Soft Gold ..... 210
Using Clamps ..... 332
Vigo on Toothache, 1514 ..... 407
Vain Veracity ..... 480

### Communicated.

### NEW JERSEY STATE DENTAL SOCIETY.

The Twenty-eight annual meeting of the New Jersey State Dental Society will be held in Asbury Park, on July 20 and 22d, inclusive.

The Committee on Exhibits is more than pleased to announce that they have again secured the Auditorium, an ideal building for our purpose. Being commodious, everything in connection with Dentistry can be accommodated under the same roof. The building will be at the disposal of the Exhibition from Monday morning, July 18th. This will give ample time to arrange exhibits prior to the convening of the Convention, at 10 o'clock on Wednesday morning. The Auditorium is situated in the centre of the block, directly facing the beach, the side enclosures being principally windows, all of which are available for ventilating purposes, which insures a cool and comfortable meeting place at all times. Several thousand dollars has been spent in making extensive repairs and alterations during the past winter, and its appointments have been made perfect. Wires for electric lighting, power, telephone and telegraphy, as well as high water pressure is at our disposal

A branch of the Asbury Park Post Office has been established in the building. Also a Bureau of Information, with a constant attendant and a private watchman, has been secured to look after the interests of the exhibitors.

The Committee can assure all who desire to exhibit that there will be a large attendance; in fact from the plans already formulated, we feel confident that this year will surpass any in the history of this Society. We trust that we shall have you with us this coming summer, and believe that it will be to your interest to secure the necessary space at an early date. Those engaging space prior to the programme going to print will be mentioned therein.

Only one restriction is made by the Committee. All goods must be covered during meeting hours.

When the desired location is secured, price of the same must be deposited, or space will not be reserved.

Exhibition tables are four feet wide, the length is marked upon the plan. Price, $1.25 per running foot for any length less than six feet. All over six feet, $1.00 per running foot. Nothing less than four feet will be sold. Exhibits may be arranged from July 18th, at 8 A. M., to July 22d, at 9 A. M. All communications as to space to be addressed to the chairman,

J. ALLEN OSMUN, M. D. S.,
588 Broad Street,
Newark, N. J.

---

### THE NATIONAL DENTAL ASSOCIATION.

The next annual meeting of the National Dental Association will be held in Omaha, commencing on Tuesday, the 30th day of August, 1898.

Attention is called to the fact that all who were members of the American Dental Association and of the Southern Dental Association at the time of the formation of the National Dental Association, are now members of the latter organization.

The Constitution, Article III, Section 5, provides as follows:

"It is hereby specially provided that all persons at present permanent members of the American Dental Association and of the Southern Dental Association, are permanent members of this Association, and entitled to all the privileges of the class to which they belonged without further action, and the Treasurer is hereby directed to transcribe their names upon the roll of membership of this Association."

The officers of the National Dental Association will leave nothing undone to make the meeting at Omaha a success, and they hope the attendance and interest in the first active annual meeting of the Association will be commensurate with its importance.

By order of
THOMAS FILLEBROWN,
President.

EMMA EAMES CHASE,
Corresponding Secretary.

---

### MEETING OF AMERICAN MEDICAL PUBLISHERS' ASSOCIATION.

The fifth annual meeting of the American Medical Publishers' Association will be held in Denver, on Monday, June 6th, 1898 (the day preceeding the meeting of the American Medical Association). Editors and publishers, as well as every one interested in Medical Journalism, cordially invited to attend and participate in the deliberations. Several very excellent papers are already assured, but more are desired. In order to secure a place on the program, contributors should send titles of their papers at once to the Secretary.

CHAS. WOOD FASSETT,
St. Joseph, Mo.

---

### THE CHICAGO DENTAL SOCIETY.

The Officers of the Chicago Dental Society for 1898-99, elected at the annual meeting, held in the Stewart Building, Tuesday Evening, April 5th, 1898; are as follows:

President, J. E. Hinkins; First Vice-President, D. C. Bacon; Second Vice-President, E. A. Royce; Recording Secretary, Elgin Ma Whinney; Corresponding Secretary, C. S. Bigelow; Treasurer, E. D. Swain; Member Board of Directors, J. G. Reid; Board of Censors, A. W. Harlan, W. V. B. Ames, C. N. Johnson.

---

### Monthly Summary.

### To Readers and Correspondents.

Communications intended for publication, and exchange journals and papers, should be addressed to the Editor of The American Journal of Dental Science, No. 845 N. Eutaw St. Baltimore, Md. All letters relating to the business of the Journal, or containing cash remittances, to be directed to Snowden & Cowman Mfg. Co., 9 W. Fayette Street, Baltimore, Md. Articles for publication should be received by the Editor at least two weeks previous to the first of the month on which the number in which it is designed for them to appear is issued.

### CONTENTS:

### SELECTIONS.

Article I.—Pathologic Conditions of the Pharynx and Contiguous Structures During Early Childhood, Prime Factors in the Etiology of Malformed Maxillæ and Irregular Teeth, Etc. Wil iam A. Mills. D. D. S ... 1

" II.—Method of Shortening the Period of Pain During the Action of Arsenical Applications .......... 5

" III.—The Drug Habit. A. W. Harlan, M. D., D. D. S...... 7

" IV.—A Clinic on a Fusible Alloy. Grant Molyneaux, D.D S. 12

" V.—Use of Screws. H. W. Arthur, D. D. S.......... 16

" VI.—Some Failures Attending the Use of Weld's Chemico-Metallic Method of Filling Root Canals. Dr. H. B. Hinman .......... 22

" VII.—Dentistry in the Twenties. D. D. Atkinson, D. D. S... 23

" VIII.—Amalgam.......... 31

" IX.—Little Things in Great Things.......... 33

### COMMUNICATED.

New Jersey State Dental Society .......... 36

The National Dental Association .......... 37

Meeting of American Medical Publishers' Association.......... 38

The Chicago Dental Society .......... 38

### MONTHLY SUMMARY.

Liquefied Air.......... 39

Dishonest Dentistry.......... 40

Pyorrhea Alveolaris.......... 42

Caries vs. Erosion.......... 42

Saucer-shaped Cavities .......... 44

Opening the Bite with Cap Fillings, Preserving the Vitality of the Pulp .......... 44

A Mysterious Stain.......... 45

To Painlessly Cure a Corn .......... 45

The Use of Non-Cohesive Gold.......... 46

Immediate Investing Material.......... 46

Taking Difficult Impressions.......... 47

Mixing Amalgam.......... 47

Dr. Evans.......... 78

☞ The American Journal of Dental Science will contain fifty pages of reading matter in each number, making a volume of about Six Hundred pages,

EXCLUSIVE OF ADVERTISEMENTS.

